# Estimates of Potential Demand for Measles and Rubella Microarray Patches

**DOI:** 10.3390/vaccines12091083

**Published:** 2024-09-23

**Authors:** Lidia K. Kayembe, Leah S. Fischer, Bishwa B. Adhikari, Jennifer K. Knapp, Emily B. Khan, Bradford R. Greening, Mark Papania, Martin I. Meltzer

**Affiliations:** The U.S. Centers for Disease Control and Prevention, 1600 Clifton Rd NE, Atlanta, GA 30329, USA; wvj1@cdc.gov (L.S.F.); bia6@cdc.gov (B.B.A.); emilysbkahn@gmail.com (E.B.K.); kax9@cdc.gov (B.R.G.)

**Keywords:** transdermal patch, vaccination, microneedle, health service needs and demand, measles vaccine, rubella vaccine

## Abstract

Global measles vaccine coverage has stagnated at approximately 85% for over a decade. By simplifying vaccine logistics and administration, the measles and rubella microarray patch (MR-MAP) may improve coverage. Clinical trials have demonstrated similar safety and immunogenicity in 9-month-old infants for MR-MAPs compared with syringe-and-needle vaccination. To aid commercialization, we present estimates of MR-MAP demand. We created a spreadsheet-based tool to estimate demand for MR-MAPs using data from 180 WHO countries during 2000–2016. Five immunization scenarios were analyzed: (1a) Supplementary Immunization Activities (SIAs) in Gavi, the Vaccine Alliance (Gavi)-eligible countries and (1b) WHO countries where preventive SIAs are routinely conducted; (2) SIAs and outbreak response immunization in all WHO countries; (3) routine immunization (RI) and SIAs in six high-burden measles countries (the Democratic Republic of the Congo, Ethiopia, India, Indonesia, Nigeria, and Pakistan); (4) RI and SIAs in six high-burden countries and Gavi-eligible countries; and (5) hard-to-reach populations. MR-MAP demand varied greatly across scenarios. Forecasts for 2025–2034 estimate from 137 million doses in hard-to-reach populations (scenario 5) to 2.587 billion doses for RI and SIAs in six high-burden countries and Gavi-eligible countries (scenario 4). When policymakers and manufacturers assess MR-MAP demand, they may consider multiple scenarios to allow for a complete consideration of potential markets and public health needs.

## 1. Introduction

Measles remains one of the leading causes of childhood morbidity and mortality worldwide, and rubella is the leading cause of preventable severe birth defects, despite the availability of a safe and effective vaccine against these diseases since the 1960s [1]. Global measles routine immunization coverage has stagnated at around 85% for over a decade. Coverage declined to approximately 81% during the COVID-19 pandemic, increasing to 83% in 2022 1]. Coverage levels falling short of 95% are insufficient to stop ongoing transmission. In 2022, there were 9,232,300 estimated measles cases worldwide [2] and 17,407 rubella cases reported to the World Health Organization [3]. In 2022, measles killed an estimated 136,200 people—mostly children under five years of age [1]. Additionally, worldwide, 117 million children currently are at risk for measles due to weakened immunization systems, delayed campaigns, and a decline in disease surveillance caused by the COVID-19 pandemic and response [4].

Increasing global immunization coverage among children in resource-constrained settings is challenging for many reasons, including the characteristics of the existing syringe-and-needle (S/N) vaccination technology. Current S/N technology requires all measles-containing vaccines (MCVs) to be consistently stored between 2 °C and 8 °C until the point of delivery. This storage requirement can be difficult to maintain in many settings. In low- and middle-income countries (LMICs), MCVs are typically procured in 10-dose vials to minimize costs due to packaging and storage requirements. The vaccine is shipped and stored in two parts (a lyophilized powder and sterile water), reconstituted at the point of delivery, and must be used within 6 h [5]. Onsite reconstitution and multidose withdrawal from the vial may create risks for contamination and diluent errors. Challenges of syringe-and needle (S/N) administration include the need for highly trained immunization staff (who are in short supply in many LMICs), disposal of sharps, risk of needle stick injury to healthcare workers, and the pain associated with injection, which might limit potential demand for vaccination. To overcome these barriers to increasing immunization coverage, alternative vaccine technologies are needed that have less strict cold chain requirements (especially at the point of delivery), require no reconstitution, are made in a single-dose format, and can be delivered without S/N.

An existing technology, the measles–rubella vaccine microarray patch (MR-MAP), can potentially address the key logistical barriers to immunization delivery in resource-constrained settings. MAPs consist of multiple microprojections (<1 mm tall) mounted on a small coin-sized patch applied to the skin by thumb pressure or with an applicator [6,7,8]. Because the penetration of the projections into the skin does not reach the nerve layer, the application of MAPs is often painless. MR-MAPs are more thermostable than the lyophilized vaccine, and no diluent or reconstitution is needed. They do not require highly trained personnel for administration, are needle-free, and do not have the risk of needle reuse. There is also the potential that MR-MAPs may not need to be kept in the cold chain, unlike the current measles vaccine technology. Recent phase 2 clinical trials have shown similar immunogenicity and safety results when comparing MR-MAPs with S/N MR vaccination in subjects as young as 9 months [8]. In those trials, there were no acute allergic reactions in either toddlers or adults. The most common vaccine-related reaction among toddlers was a mild local reaction at the MR-MAP application site, with 50 toddlers (83%) having such a mild local reaction, compared with 18 toddlers (30%) at the placebo-MAP application site. Adults in the MR-MAP group had almost the same rate of local solicited adverse events as adults in the placebo-MAP group. There were no related severe or serious adverse events [8]. In infants, vaccination with MR-MAPs resulted in 93% (95% CI 83.0–97.2) seroconverting to measles and 100% (93.8–100) seroconverting to rubella. Among infants vaccinated with S/N technology, 90% (79.2–95.2) and 100% (93.9–100) seroconverted to measles and rubella, respectively [8]. The WHO has produced a MR-MAP target product profile to document the desired features for use in LMICs [9].

While the potential public health benefit of MR-MAPs is clear, the business case for commercializing MR- MAPs is less so, especially for vaccine manufacturers who already have MCV products on the market. We present ten-year estimates of potential demand for MR-MAPs in five scenarios, each of which includes a different mix of countries and vaccination scenarios. These scenarios and the spreadsheet-based tool used to produce them can aid policymakers, donors, and vaccine manufacturers in making crucial decisions regarding investment in the commercialization of MR-MAPs. Our estimates extend previous work [10] by using a methodology for estimating future potential demand by statistical, regression-based extrapolation. We used country-specific coverage rates of growth, stagnation, or decline, based on historical data trends. Further, we provide a spreadsheet tool allowing users to update the data included and examine potential demand under alternate scenarios of their choosing.

## 2. Methods

### 2.1. Tool Overview

We developed a spreadsheet-based “Tool for Estimating the Potential Demand of Measles and Rubella Microarray Patches” (hereafter, “the Tool”) to estimate MR-MAP potential demand (see the Spreadsheet Tool as a Supplement.). Potential demand estimates were based on data from 180 WHO countries with the necessary data variables (14 WHO member states for which there were no data available to complete the analysis were omitted from the list (Andorra, Antigua and Barbuda, Cook Islands, Dominica, Kiribati, Marshall Islands, Monaco, Nauru, Niue, Palau, Saint Kitts and Nevis, San Marino, Seychelles, and Tuvalu)). The list of variables used in the Tool is provided in Table 1 (including where country-specific values can be found).

The Tool includes five population-level immunization scenarios for routine immunization (RI) and Supplementary Immunization Activities (SIAs), as follows (see Appendix A for the list of countries by scenario):

(1a) Supplementary Immunization Activities (SIAs) in the Gavi, the Vaccine Alliance (Gavi)-eligible countries as listed in 2024 (inclusive of countries in transition phases).
(1b) WHO countries routinely holding large-scale SIAs.
(2) SIAs and outbreak response immunization in all WHO countries.
(3) SIAs and routine immunization in six high-burden measles countries (the Democratic Republic of the Congo, Ethiopia, India, Indonesia, Nigeria, and Pakistan) (the six high-burden countries were selected by the then Measles and Rubella Initiative (M&RI) formed by the American Red Cross, CDC, United Nations Foundation, UNICEF and WHO; the M&RI was replaced, in 2023, by the Measles and Rubella Partnership (M&RP) which includes new partners, Gavi and the Bill and Melinda Gates Foundation, and is under the umbrella of the Immunization Agenda 2030).
(4) SIAs and routine immunization in six high-burden countries and Gavi-eligible countries.
(5) Hard-to-reach (hard-to-reach populations of any given country are defined as the population represented by the difference between the coverage needed to attain measles herd immunity (95%) and the WHO/UNICEF National Immunization Coverage Estimates for the first dose of measles-containing vaccine in a given year) populations (niche market).

### 2.2. Base Case and Alternate Adoption Scenarios

For the base case, for each scenario it is assumed that MAPs will replace all MCV1 and MCV2 doses forecasted to be administered by the existing S/N technology (i.e., 100% adoption of all vaccinations administered). Note that for Scenario 5 (the hard-to-reach populations), because there are no existing coverage data, we assume in the base case that 100% of this population will be vaccinated with MAPs. For a variety of reasons, including price and production capacity constraints, it is likely that there will not be an instantaneous replacement of S/N by MR-MAPs. Therefore, we estimated the partial adoption of the technology in 50% and 90% of the estimated baseline potential demand of MR-MAPs over 10 years for each scenario. These estimates do not represent a confidence interval but alternate outcomes of the degree of acceptance of the new vaccination technology.

### 2.3. Adjusting the Scenarios

A user of the Tool can adjust almost all the input values (Table 1) and other aspects. For example, a user can select the starting year of the forecast (2025 to 2030) and the duration (5 or 10 years) and adjust the vaccine wastage rate for routine immunization and Supplementary Immunization Activities in each scenario. The user can also change the historical and projected MCV coverage of RI and SIAs for each of the 180 countries (see Appendix F).

### 2.4. Data Sources

For the 180 countries included in the Tool, we used historical routine immunization coverage data from 2000 to 2016 on measles-containing vaccines/rubella-containing vaccines (MCVs/RCVs) from the WHO UNICEF Estimates of National Immunization Coverage (WUENIC) database (Table 1).

We used historical data on MR SIAs from the WHO SIA database (2000–2016), and we complemented these data with country-specific Expanded Programme on Immunization (EPI) data when needed. Countries were categorized using the 2017 World Bank per capita Gross National Income classification system [15]. Country-specific demographic information was taken from the World Population Prospects 2017, published by the United Nations Department of Economic and Social Affairs (UNDESA) [16]. Vaccine wastage rates for routine immunization and SIAs came from data from various WHO countries [11,12,13,14].

### 2.5. Forecast of RI Demand

We assume that the rubella-containing vaccine (RCV) will be introduced with an MR SIA for children under 15 in all countries prior to the public availability of MR MAPs; in this study, this was assumed to occur by 2025, the first year of estimates in the vaccine potential demand forecasting Tool. Countries introduce the rubella vaccine in a combined measles–rubella formulation, which is then used for all MR vaccination activities. Therefore, the estimated RCV coverage equals the MCV coverage for both the first and second doses.

**Figure 1:**
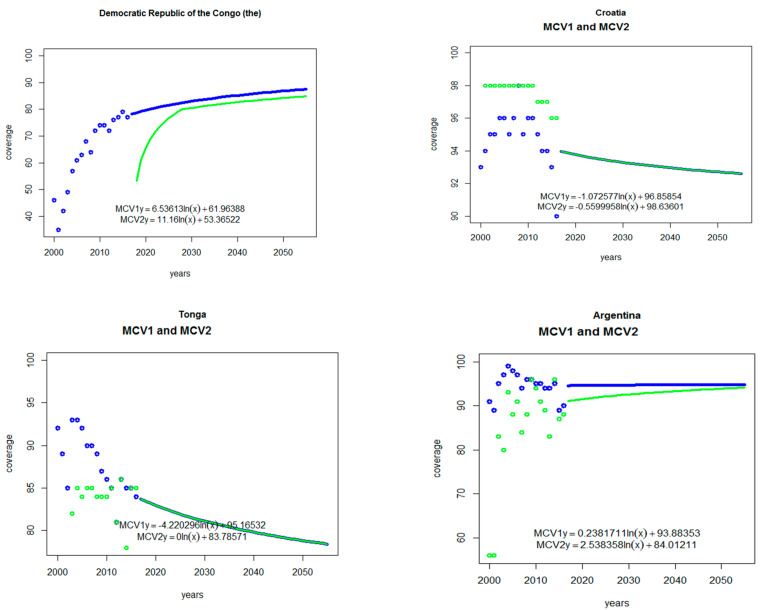
Examples of MCV1 and MCV2 historical coverage and estimated future coverage (2000–2055) Notes: Blue and green dots represent the historical coverage rates for the measles-containing vaccine first dose (MCV1) and measles-containing vaccine second dose (MCV2), respectively. The solid blue and green lines represent the future estimates of such coverage based on extrapolations from regression analyses. See text for additional details. The equations in each graph are logistic regressions used to fit the historical data and subsequently used to estimate future potential demand by extrapolating forward, producing the solid line shown.

Following the methodology of Winter et al. [17], we estimated the potential demand for MR-MAP for each country, using country-specific historical coverage data of measles and rubella RI and SIAs. We first used natural logarithmic regression to fit the 2000–2016 historical MCV1 coverage data for each country. We then estimated future potential demand year by year (2017–2055), using the equation set out in Appendix E. For each country and each future year, the equation combines the target population, the extrapolated MCV1 coverage, and the wastage rate. For each country, for each year (both historical and estimates of future coverage), the data are available in the Tool (see Table labeled “Contents Variables List”). Figure 1 provides four country-specific examples of estimates of future MCV routine immunization coverage based on historical MCV coverage data. As shown in Figure 1, using the regression equations to extrapolate forward resulted in estimates of future coverage increasing at a decreasing rate (i.e., diminishing returns). For 22 (European region: Georgia, Kyrgyzstan, Moldova, Italy, Malta, Russian Federation, Serbia, Slovenia, Spain, Macedonia; Eastern Mediterranean region: Djibouti, Jordan, Pakistan, Yemen, Libya, Oman, Saudi Arabia, UAE; Western Pacific region: Mongolia, Philippines, Fiji, Malaysia) of the 180 countries, MCV1 coverage did not follow a natural logarithmic function, and we averaged their most recent coverage data. We extrapolated forward by holding each respective average coverage rate constant. A user of the Tool can adjust the estimates of potential future coverage (and thus automatically update or alter estimates of demand) by adding in their own estimates of future coverage (Appendix F).

We used several approaches to estimate the future coverage of the second dose of the measles-containing vaccine (MCV2). For countries with 4 or more years of MCV2 coverage data, we applied the natural logarithmic function process as described for the MCV1 doses. For countries with 0 to 3 years of MCV2 data, we used the average fit (slope) from similar countries, as classified by the World Bank’s income classification [18]. For example, for an African country in the low-income World Bank classification group, we used the slope derived from all countries in the same category with 4 or more years of MCV2 data.

We then estimated MCV2 coverage as a fixed percentage of MCV1 coverage. The fixed percentage of MCV1 coverage percentage difference was calculated based on region-income level-specific MCV1-MCV2 differences in countries with at least 4 years of MCV2 data (Appendix B). For example, a low- or middle-income country in the Africa region had MCV2 coverage 32% lower than its MCV1 value in the year of MCV2 introduction.

Forecasted coverage levels were used to estimate future doses needed. We set the upper limit of future MR coverage estimates to 99% and further assumed that MCV2 could not exceed MCV1 (detailed explanation in Appendix C). In total, 360 individual equations were created to estimate MCV coverage (one equation per country for each MCV dose provided through routine immunization).

### 2.6. Forecast of SIA Demand

The timing of future SIAs (2017 onward) was set as the year when the number of measles-susceptible individuals reached the size of a birth cohort in a particular country (the susceptibility threshold) or four years since the last SIA, whichever occurred first. All preventive SIAs target children under five years of age, with a lower bound age of nine months. Future SIA coverage is based on the country’s average historical coverage. To account for potential improvement in the planning and implementation of SIAs, SIA coverage was increased by 10% of the difference between the previous SIA coverage estimate and 100% for three subsequent SIAs and held constant through 2055.

To ensure the reality of SIA implementation, we added a cessation rule aligning with the measles vaccines WHO position paper [19] “Cessation of SIAs should be considered only when >90–95% immunization coverage has been achieved at the national level for both MCV1 and routine MCV2 as determined by the most accurate means available for a period of at least 3 consecutive years”.

The following additional three criteria had to be met for a country to stop SIAs:

1. Two doses of MCVs were part of the national EPI schedule for more than 5 years;
2. RCVs were introduced with an MR SIA for those under 15 years and at least one additional follow-up campaign; and
3. The susceptibility threshold was not reached within 8 years after the previous SIA.

### 2.7. Potential Total Demand Estimation

For each of the 180 countries included in the Tool, we estimated future MCV1 and MCV2 coverage and future SIA timing and coverage. Examples of future SIA timing and coverage are shown in Appendix D.

RI and SIA coverage were converted into MR doses needed by multiplying these values by the size of the target population while accounting for vaccine wastage rates (Appendix E). These calculations were applied to all countries of each of the five scenarios for 10 years, from 2025 to 2034, and the number of doses needed was summed by scenario.

## 3. Results

### 3.1. Base Case

**Figure 2:**
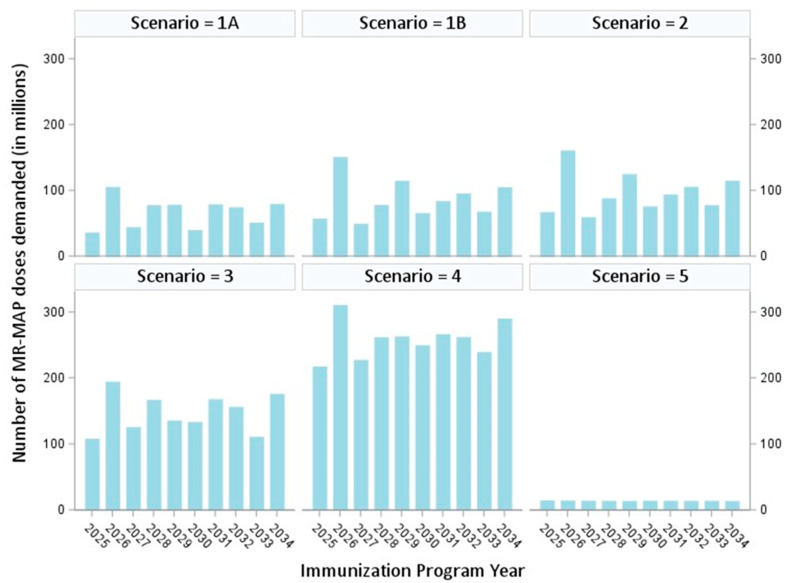
MR-MAP potential demand by estimation scenario and by year (2025–2034) Notes: The measles–rubella microarray patch potential demand scenarios are for (1A) SIAs in Gavi, the Vaccine Alliance (Gavi)-eligible countries; (1B) SIAs in all WHO countries; (2) SIAs and outbreak response immunization; (3) SIAs and routine immunization in six high-burden measles countries: the Democratic Republic of the Congo, Ethiopia, India, Indonesia, Nigeria, and Pakistan; (4) SIAs and routine immunization in six high-burden countries and in Gavi countries; and (5) hard-to-reach populations. Note that for Scenario 5, because there are no existing coverage data, we assume in the base case that 100% of this population will be vaccinated with MAPs.

The base case results represent the estimated MR-MAP potential demand when MAPs fully replace S/N technology (i.e., 100% adoption). As shown in Table 2, MR-MAP potential demand varies from 137 million doses (Scenario 5: hard-to-reach populations) to 2587 million doses (Scenario 4: SIAs and routine immunization in six high-burden countries and Gavi-eligible countries). As shown in Figure 2, MR-MAP potential demand varies over time for any given scenario (Table 2 contains the 10-year cumulative totals for each scenario). The driving factor behind the year-to-year fluctuations shown in Figure 2 is the occurrence of SIAs in countries with large under-five populations (e.g., Ethiopia, the Democratic Republic of the Congo, Indonesia, Nigeria and Pakistan). For example, for Ethiopia, we assumed SIAs would take place in the years 2027 and 2031, which would consume 23,505,000 and 24,028,000 MR-MAP doses, respectively, compared to the years between 2025 and 2039 when SIAs do not take place and when annual potential demand varies between 7,042,000 and 7,396,000 doses. Note that for Scenario 5 (hard-to-reach populations), in the absence of historical coverage data for this group, we assumed in the base case that 100% of this population will be vaccinated with MAPs, with no SIAs. This results in a steady year-to-year demand, with no fluctuations.

### 3.2. Alternate Estimates

Table 2 shows the base case estimation and alternate results for 50% and 90% adoption of the new technology. When considering lower levels of adoption, such as assuming 50% of MCV1 and MCV2 doses are administered by MR-MAP technology, the 10-year estimates of MR-MAP potential demand declines to between 68 and 123 million doses (Scenario 5: hard-to-reach populations) and between 1294 and 2328 million doses (Scenario 4: SIAs and routine immunization in six high-burden countries and Gavi-eligible countries).

## 4. Discussion

Our 10-year estimates of potential demand for MR-MAPS, assuming a 100% replacement of S/N technology, ranged from 137 million doses in hard-to-reach populations to 2.587 billion doses for RI and SIAs (Table 2). Reducing the assumption to 50% replacement of doses administered by S/N yielded 10-year estimates of MR-MAP potential demand ranging from 137 million doses to 1294 million doses. Of potentially equal importance when considering potential demand over 10 years is the estimated year-to-year fluctuations in potential demand. In some scenarios, such as Scenario 2 (SIAs and outbreak responses in WHO countries), potential demand in a given year can almost double or be halved compared to the previous year. The scenarios with larger 10-year total potential demands (e.g., Scenarios 3 and 4) show less frequent large year-to-year fluctuations.

The WHO estimated MR-MAP potential demand and presented three eleven-year estimates of potential demand for 2030 to 2040, ranging from 1.2 million doses (regional MR-MAP pilots) to 4.05 billion doses (for RI and SIAs) [10]. The latter was revised to 1.46 billion doses when the WHO applied assumptions of country adoption and market penetration, which are lower than our MAP demand estimates for RI and SIAs but are within the range of our five scenarios. The main reason for the difference between our estimates is that the WHO estimates were produced assuming a uniform annual growth of 3% in MCV coverage in each country. As noted earlier, we used country-specific coverage growth rates, with some countries growing at faster rates than others, others having a declining growth rate over time, and others having stagnated. Our methodology also included, for those estimated to have growth over time, a decreasing rate of increase over time. Further, unlike the WHO estimates, we did not have any scenario in which all countries simultaneously adopted MR-MAPs, which limited our estimates of potential demand. We excluded an all-countries-simultaneously-adopt scenario because we think there may be both notable lags in willingness to adopt MR-MAPs and delays in building production capacity to achieve such a scenario realistically.

Our estimates of MR-MAP potential demand are limited in a few ways. We did not include the COVID-19 pandemic-related reductions in coverage in our models (Figure 1). We considered such reductions in coverage to be anomalies not indictive of non-pandemic demand. While universal rubella vaccine introduction may not be complete by 2025, the vast majority of the global population should have the rubella vaccine prior to the public availability of MR-MAPs; by the end of 2022, 175 of 194 countries were already using MR vaccines [3], with additional countries already preparing for rubella vaccine introduction. Our estimates also do not incorporate the effects of the cost of MR-MAPs, because reliable product costs are not yet readily available. Two papers report the cost-effectiveness of MR-MAPs. One paper [20] used a range of USD 0.95 to 1.19 (2010 USD)/per MR-MAP. Another paper [21] used, for low-income countries, costs adjusted for wastage ranging from USD 1.30 to 2.95 (2020 USD). This latter range was compared to the USD 0.86 for the Gavi-priced 5-dose vials for the MR vaccine [21]. For both papers, however, MR-MAP costs are speculative, as there are no published costs for MR-MAP production and marketing.

Two companies, one in Australia and one in the U.S., are setting up pilot production lines which may each produce up to 10 million MR-MAP doses/year [22,23]. Reaching such a production goal may be some years in the future, as both companies need to conduct safety-and-efficacy Phase III trials and have their facilities inspected and certified. Creelman et al. have detailed the barriers and problems which must be overcome to achieve full-scale production of MR-MAPs [24]. In the initial years of MR-MAP production and use, S/N is likely to be less expensive per dose administered. The impact on the potential demand for MR-MAPs caused by higher prices than S/N technology can be examined by considering the 50% and 90% demand forecasts presented in Table 2, as well as using the Tool to further examine additional alternative adoption rates. An additional important limitation is, from the vaccine manufacturer’s business perspective, the potential demand for MR-MAPs essentially replacing the existing demand for MCVs administered via S/N. Thus, from the vaccine manufacturer’s point of view, much of the estimated MR-MAP demand presented here represents a “cannibalization” of an existing market. Such cannibalization may reduce existing MR vaccine producers’ interest in adopting the MAP technology. Finally, we did not allow for the fact that it may take time for production capacity to grow to the levels needed to meet some of the calculated potential demand estimates. Allowing for such production constraints would require currently unavailable data on production capacities and the growth of such capacities. In the Tool, the estimates presented here can be adjusted downward to account for the assumed impact of these limitations.

## 5. Conclusions

MR-MAPs could revolutionize vaccine delivery, making MR vaccines more accessible to the approximately 22 million children who missed their first measles vaccine dose in 2022 [25]. The large-scale adoption of MR-MAPs, however, may need to overcome the already noted potential problems of price differentials (i.e., existing vaccination technologies may be notably cheaper, at least initially) and “market cannibalization”. Policymakers may wish to use our estimates to help consider various options to spur potential demand for MR-MAPs. Such “market-enhancing” options might include strategies such as purchase orders guaranteeing the purchase of a minimum number of MR-MAPs and/or agreements to subsidize the purchase of a given number of MR-MAPs. Such strategies would likely require building a consensus between potential users (e.g., Ministries of Health), regulatory authorities, manufacturers, and donor organizations. Considerations and estimates provided by the Tool may help build a global consensus regarding the benefits of producing large numbers of MR-MAPs to improve global MCV coverage and the public health of tens of millions of children and adults around the world.

## Figures and Tables

**Table 1:** Variables used to estimate measles and rubella microarray patch (MR-MAP) potential demand.

Variable *	Description	Location of Values in the Tool ^†^
MR-MAP wastage rate	Vaccine wastage rate by scenario, 15–30%, scenario-dependent ^‡^	MAP characteristics tab
Global annual MAP potential demand for outbreak responses	Baseline value of global annual MR-MAP potential demand for outbreak response used in Scenario 2, assumed: 10 million	Adjusting MAP demand tab
Adoption rate of MR-MAPs	MR-MAP assumed estimated potential demand adjusted for partial adoption of the new technology; assumed baseline range: 50%, 75%, 90%	Adjusting MAP demand tab
MCV1	Estimated future coverage for the first dose of measles-containing vaccine (180 country-specific; 2025–2055)	MCV1 tab
MCV2	Estimated future coverage for the second dose of measles-containing vaccine (180 country-specific; 2025–2055)	MCV2 tab
Historical MCV1	Coverage for the first dose of measles-containing vaccine (WUENIC country-specific estimates, for 180 countries, 2000–2016) **	Historical MCV1 tab
Historical MCV2	Coverage for the second dose of measles containing vaccine (WUENIC country-specific estimates, for 180 countries, 2000–2016) **	Historical MCV2 tab
SIAs	Tool-estimated historical and future coverage of MR Supplementary Immunization Activities (SIAs) (180 country-specific; 2025–2055) ^††^	SIA tab
SIA age	Upper age limit of 5 years for SIAs	SIA age tab
Surviving infants	Number of surviving infants by year per country, for 180 countries (UNDESA; 2025–2035)	SurvInf tab
Under 12 months population	Number of children under 12 months of age by year per country, for 180 countries (UNDESA; 2025–2055)	Population < 12 mo tab
Under 5 years population	Number of children under 5 years of age by year per country, for 180 countries (UNDESA; 2025–2055)	Population < 59 m tab
Gavi countries	List of 54 Gavi-eligible countries in 2024	Gavi countries tab
WHO countries	List of 180 WHO countries	WHO states tab
Six countries	The six high-burden countries are the DRC, Ethiopia, Nigeria, India, Indonesia, and Pakistan, selected by the Measles and Rubella Partnership (M&RP)	MRI tab

* MR-MAP = measles and rubella microarray patch; MCV1 = measles-containing vaccine first dose; MCV2 = measles-containing vaccine second dose; WUENIC = WHO/UNICEF Estimates Of National Immunization Coverage; SIA = supplementary immunization activity; UNDESA = The United Nations Department of Economic and Social Affairs; Gavi = Gavi, the Vaccine Alliance; WHO = World Health Organization; DRC = Democratic Republic of the Congo. ^†^ “Tool” refers to the Excel spreadsheet Tool provided as Supplementary Information. The “Location in Tool” column refers to specific sheets (“Tabs”) in the Tool. Each Tab in the Tool contains the values used and the source of such values. ^‡^ See “Data sources” in the main text for an explanation of the source of these wastage rates. References [11,12,13,14]. ** Available online: https://www.who.int/teams/immunization-vaccines-and-biologicals/immunization-analysis-and-insights/global-monitoring/immunization-coverage/who-unicef-estimates-of-national-immunization-coverage (accessed on 19 July 2024). ^††^ Available online: WHO/IVB Database workbook containing summary of Supplementary Immunization Activities (SIAs). https://immunizationdata.who.int/global?topic=Additional-datasets&location= (accessed on 19 July 2024).

**Table 2:** Estimates of potential demand for measles and rubella microarray patches (MR-MAPs) in millions of doses for 10 years, 2025 to 2034.

Adoption Scenarios *	MR-MAP Usage	Target Population: Cumulative Population over 10 Years (in Millions) ^†^	Cumulative Estimated Potential Demand of MR-MAPs, 2025–2034 (Millions of Doses)	
			Base Case (Assuming 100% Adoption) ^‡^	Assuming Lower Levels of Adoption (50% to 90% Transition from S/N to MAPs) ^¶^
Scenario 1A	Supplementary Immunization Activities in Gavi-eligible countries	2341	663	331–596
Scenario 1B	Supplementary Immunization Activities in WHO countries	2653	866	433–779
Scenario 2	Supplementary Immunization Activities and outbreak responses-WHO countries	2653 + 10 million doses ^§^	966	483–869
Scenario 3	Supplementary Immunization Activities and routine immunization in six high-burden countries	2651	1471	736–1324
Scenario 4	Supplementary Immunization Activities and routine immunization in six high-burden countries and GAVI-eligible countries	4421	2587	1294–2328
Scenario 5	Hard-to-reach populations	137	137	68–123

Notes: * Description of scenarios: (1a) supplementary immunization activities (SIAs) in Gavi, The Vaccine Alliance (Gavi)-eligible countries (inclusive of countries in transition phases in 2018 and (1b) in WHO countries routinely holding large-scale SIAs; (2) in SIAs and outbreak response immunization in all WHO countries; (3) in SIAs and routine immunization in six countries with high measles burden (Democratic Republic of the Congo, Ethiopia, India, Indonesia, Nigeria, and Pakistan); (4) in SIAs and routine immunization in six high-burden countries and Gavi-eligible countries; (5) and in hard to reach populations. Scenario 5 represents the additional population of the 180 WHO countries needing to be vaccinated to reach 95% MCV1 coverage for each of the countries in the analysis. Note that for Scenario 5, because there are no existing coverage data, we assume in the base case that 100% of this population will be vaccinated with MAPS. ^†^ The total target population represents the cumulative population targeted for RI (MCV1 and MCV2) and SIAs from 2025 to 2034. ^‡^ 100% adoption here means microarray patches are assumed to replace all MCV1 and MCV2 doses forecasted to be administered by the existing needles-and-syringe technology. ^¶^ Estimates of MR MAPs potential demand if MR-MAPs replace 50% and 90% of MCV doses currently administered by the existing needles-and-syringe technology (routine immunization and SIAs). ^§^ Scenario 2 includes an estimate of 10 million doses of MR-MAP needed for outbreak response at the global level.

## Data Availability

Publicly available data sources are cited in the manuscript and references. Archived versions of the data and additional calculated projection data are included in the Tool.

## Supplementary Materials

The following supporting information can be downloaded at: https://www.mdpi.com/article/10.3390/vaccines12091083/s1. The Spreadsheet Tool as a Supplement.

## Appendix A. Cumulative 10-Year Target Population Size by Country by Scenario

**Country**	**Scenario 1A Population (In 000)**	**Scenario 1B Population (In 000)**	**Scenario 3 Population (In 000)**	**Scenario 4 Population (In 000)**	**Scenario 5 Population (In 000)**
Afghanistan	50,306	50,306		61,583	2605
Albania					
Algeria					
Angola		64,743			4764
Argentina					22
Armenia					
Australia					
Austria					20
Azerbaijan					
Bahamas					2
Bahrain					
Bangladesh	122,479			148,775	9
Barbados					2
Belarus					
Belgium					
Belize					
Benin	20,094	20,094		24,845	1144
Bhutan					
Bolivia					
Bosnia and Herzegovina					14
Botswana					
Brazil					
Brunei Darussalam					
Bulgaria					10
Burkina Faso	36,846	36,846		45,560	523
Burundi	21,658	21,658		26,795	
Cambodia	15,022	15,022		18,338	431
Cameroon	40,951	40,951		50,526	1006
Canada					196
Cape Verde					
Central African Republic	7795	7795		9622	744
Chad	30,524	30,524		37,767	2531
Chile					76
China					
China, Hong Kong Special Administrative Region					675
China, Macao Special Administrative Region					68
Colombia					149
Comoros	1195	1195		1470	19
Congo	11,352	9194		11,352	219
Costa Rica					20
Côte d’Ivoire	44,133	44,133		54,533	2725
Croatia					5
Cuba					
Cyprus					8
Czech Republic					
Democratic People’s Republic of Korea	15,183			18,467	
Democratic Republic of the Congo	171,096	171,096	211,750	211,750	4938
Denmark					30
Djibouti	906	906		1105	30
Dominican Republic		8835			185
Ecuador		14,280			183
Egypt					407
El Salvador					25
Equatorial Guinea		2152			285
Eritrea	7533			9277	
Estonia					2
Ethiopia	149,947	149,947	183,748	183,748	6279
Federated States of Micronesia					3
Fiji					2
Finland					0
France					242
Gabon		2571			146
Gambia	4016			4959	
Georgia					6
Germany					
Ghana	40,232	40,232		49,481	250
Greece					
Grenada					
Guatemala		18,939			440
Guinea	22,103	22,103		27,273	1718
Guinea-Bissau	2986	2986		3683	95
Guyana					
Haiti	10,663	10,663		13,023	793
Honduras		8582			196
Hungary					
Iceland					1
India			1,290,390	1,290,390	6528
Indonesia		204,516	249,566	249,566	7025
Iran (Islamic Republic of)					
Iraq		63,688			5151
Ireland					
Israel					
Italy					313
Jamaica					12
Japan					23
Jordan					
Kazakhstan					
Kenya	74,170	74,170		91,239	2081
Kuwait					
Kyrgyzstan	5696			6960	
Lao People’s Democratic Republic	7833	6422		7833	194
Latvia					3
Lebanon		3183			83
Lesotho	2571	2571		3143	21
Liberia	7999	7999		9870	350
Libyan Arab Jamahiriya					
Lithuania					4
Luxembourg					
Madagascar	43,214	43,214		53,262	2900
Malawi	33,943	33,943		41,864	493
Malaysia					
Maldives					
Mali	39,296	39,296		48,681	1852
Malta					4
Mauritania	7149	7149		8818	305
Mauritius					0
Mexico					0
Mongolia					0
Montenegro					19
Morocco					0
Mozambique	57,737	57,737		71,429	1061
Myanmar	40,055	40,055		48,884	553
Namibia		3296			84
Nepal	23,477	23,477		28,480	178
Netherlands					
New Zealand					
Nicaragua					
Niger	59,118	59,118		73,808	2185
Nigeria	439,216	355,901	440,357	440,357	31,743
Norway					
Oman					
Pakistan	224,766	224,766	274,998	274,998	15,720
Panama					17
Papua New Guinea	12,941	10,544		12,941	294
Paraguay					43
Peru		25,283			378
Philippines		108,950			1971
Poland					
Portugal					
Puerto Rico					322
Qatar					
Republic of Korea					
Republic of Moldova					6
Romania					98
Russian Federation					
Rwanda	16,751			20,587	
Saint Lucia					
Saint Vincent and the Grenadines					
Samoa					8
Sao Tome and Principe	323	323		399	0
Saudi Arabia					
Senegal	27,320	27,320		33,701	475
Serbia					39
Sierra Leone	11,277	11,277		13,853	302
Singapore					
Slovakia					0
Slovenia					1
Solomon Islands	988			988	
Somalia	32,045	32,045		39,759	3388
South Africa		49,049			2406
South Sudan	21,361	21,361		26,337	3732
Spain					
Sri Lanka					
Sudan	65,013	65,013		80,216	677
Suriname					4
Swaziland		1644			12
Sweden					
Switzerland					
Syrian Arab Republic	27,545	22,468		27,545	1690
Tajikistan	10,594			12,978	
Thailand					
The former Yugoslav Republic of Macedonia					1
Timor-Leste		2209			69
Togo	12,612	12,612		15,568	278
Tonga					4
Trinidad and Tobago					6
Tunisia					
Turkey					
Turkmenistan					
Uganda	91,863	91,863		113,665	1735
Ukraine					1153
United Arab Emirates					
United Kingdom					
United Republic of Tanzania	113,951			141,069	
United States of America					1302
Uruguay					
Uzbekistan					
Vanuatu		350			34
Venezuela (Bolivarian Republic of)		25,643			310
Viet Nam					
Yemen	38,249	38,249		46,795	2308
Zambia	34,112	34,112		42,213	511
Zimbabwe	23,410	23,410		28,748	132

## Appendix B. Average Percentage Difference between MCV1 and MCV2 Coverage at the Year of Introduction of MCV2

**Table:**

WHO Region Countries	Low- and Lower-Middle Income Countries	Upper-Middle- and High-Income Countries
AFR	32.2%	16.1%
AMR	10.8%	24.4%
EMR	20.3%	N/A
SEAR	40.1%	N/A
WPR	22.3%	N/A

The work of LK Kayembe and JL Kriss derived from the 2016 revision of the WHO/UNICEF Estimates Of National Immunization Coverage and the corresponding historical World Bank Country and Lending Groups can be found at https://datahelpdesk.worldbank.org/knowledgebase/articles/906519-world-bank-country-and-lending-groups (accessed on 5 September 2024). Data file is: https://datacatalogfiles.worldbank.org/ddh-published/0037712/DR0090754/OGHIST.xlsx (accessed on 5 September 2024).

## Appendix C. Relative Future MCV1 and MCV2 Coverage Estimation Methodology

At any given coverage level, the difference between MCV1 and MCV2 is subject to capping; in other words, the minimum difference between MCV1 and MCV2 varies depending on the value of MCV1 coverage. All coverage caps were determined by the median of minimum differences between MCV1 and MCV2 coverage of countries in each World Bank income group. For low- and middle-income countries with MCV1 coverage below 80%, the minimum difference is 4%; for countries with MCV1 coverage from 80% to 89%, the value is 3%. MCV2 coverage could equal MCV1 coverage when the latter is 90% or higher. Similarly, for high-income countries, MCV1 and MCV2 coverages could be equal.

## Appendix D. Supplemental Immunization Activity (SIA) Interval and Estimated Coverage for Select Countries

Country	SIA interval	SIA Coverage
DRC	4-year interval	95.3 (2028) to 95.7 (2032)
Guinea	3-year interval	95.9 (2026), 95.9 (2029), 95.9 (2032)
CAR	2-year interval	93.7 (2026), 93.7 (2028), 93.7 (2030), 93.7 (2032)

## Appendix E. Example of Estimation of Future Potential Demand for Measles Vaccine (Detailed Calculation)

Data for the DRC for the year 2028:

MCV1: 82.5%; RI wastage rate: 30%; surviving infant population size of 3,896,524.

MCV1 doses needed for 2028 for DRC (MAP1):

$$MAP1=Target\;population\times MCV1\;coverage\times wastage\;rate\;=3,896,524\times 82.5\%\times \frac{100}{100-30}\;=4,592,332$$

## Appendix F. Notes on Altering Estimates of Future Potential Demand

As described in the main text, we estimated future potential demand by extrapolating historical measles vaccination coverage for each of the 180 countries included in our estimations. We first used natural logarithmic regression to fit the 2000–2016 historical measles-containing vaccine first dose (MCV1) coverage data for each country. The results of such estimations are available in the Tool, in the Tabs labeled “MCV1” and “MCV2”.

The calculated future demand was then estimated, year by year, using the equation set out in Appendix E. For each country and each future year, the equation combines the target population, the extrapolated MCV1 coverage and the wastage rate. For each country, for each year (both historical and estimates of future coverage), the data are available in the tool (see Table labeled “Contents Variables List” for guidance as to where each dataset is in the tool).

## Appendix G. Altering the Estimates of Future Potential Demand

The Tool (MR MAP tool v1.0 FINAL.xlsm) can be accessed at https://www.mdpi.com/article/10.3390/vaccines12091083/s1. Users of the tool can adjust the estimates of potential future coverage (and thus automatically update/alter estimates of demand) by adding in their own estimates of future coverage. Such estimates need to be performed “on the sides”, with the results being placed in the appropriate country and year (in the Tab labeled “MCV1”).

Estimations of future potential demand for MCV2 can be similarly altered (but attention needs to be paid to the “Cessation Rules”—as described in the main text).
